# Changes in the ruminal fermentation and bacterial community structure by a sudden change to a high-concentrate diet in Korean domestic ruminants

**DOI:** 10.5713/ajas.18.0262

**Published:** 2018-07-26

**Authors:** Mingyung Lee, Sinyong Jeong, Jakyeom Seo, Seongwon Seo

**Affiliations:** 1 Division of Animal and Dairy Sciences, Chungnam National University, Daejeon 34134, Korea; 2 Department of Animal Science, Pusan National University, Miryang 50463, Korea

**Keywords:** High Concentrate Diet, Korean Domestic Ruminants, Fermentation Characteristics, Bacterial Diversity, Relative Population

## Abstract

**Objective:**

To investigate changes in rumen fermentation characteristics and bacterial community by a sudden change to a high concentrate diet (HC) in Korean domestic ruminants.

**Methods:**

Major Korean domestic ruminants (each of four Hanwoo cows; 545.5±33.6 kg, Holstein cows; 516.3±42.7 kg, and Korean native goats; 19.1±1.4 kg) were used in this experiment. They were housed individually and were fed *ad libitum* with a same TMR (800 g/kg timothy hay and 200 g/kg concentrate mix) twice daily. After two-week feeding, only the concentrate mix was offered for one week in order to induce rapid rumen acidosis. The rumen fluid was collected from each animals twice (on week 2 and week 3) at 2 h after morning feeding using an oral stomach tube. Each collected rumen fluid was analyzed for pH, volatile fatty acid (VFA), and NH_3_-N. In addition, differences in microbial community among ruminant species and between normal and an acidosis condition were assessed using two culture-independent 16S polymerase chain reaction (PCR)-based techniques (terminal restriction fragment length polymorphism and quantitative real-time PCR).

**Results:**

The HC decreased ruminal pH and altered relative concentrations of ruminal VFA (p<0.01). Total VFA concentration increased in Holstein cows only (p<0.01). Terminal restriction fragment length polymorphism and real-time quantitative PCR analysis using culture-independent 16S PCR-based techniques, revealed rumen bacterial diversity differed by species but not by HC (p<0.01); bacterial diversity was higher in Korean native goats than that in Holstein cows. HC changed the relative populations of rumen bacterial species. Specifically, the abundance of *Fibrobacter succinogenes* was decreased while *Lactobacillus* spp. and *Megasphaera elsdenii* were increased (p<0.01).

**Conclusion:**

The HC altered the relative populations, but not diversity, of the ruminal bacterial community, which differed by ruminant species.

## INTRODUCTION

Ruminants obtain energy and essential nutrients through a complicated symbiotic relationship with the rumen microbiome. The rumen bacterial community is affected by the type of feed ingested by the host ruminants [1]. Changes in the bacterial community in the rumen can significantly affect the health and productivity of host ruminants [2]. In order to improve the productivity of livestock ruminants, a high forage diet is commonly switched to a high concentrate diet (HC), which alters the rumen ecosystem. A HC increases the number of lactic acid producers, such as *Streptococcus bovis* (*S. bovis*) and *Lactobacillus* spp., due to its high level of non-structural carbohydrates (NSC) [3]. A HC also increases the number of lactic acid utilizers, for example, *Megasphaera elsdenii* (*M. elsdenii*), *Selenomonas ruminantium* (*S. ruminantium*), *Veillonella parvula* [3], it drastically decreases the number of fiber-degrading bacteria such as *Fibrobacter succinogenes* (*F. succinogenes*) and *Ruminococcus* spp. mainly due to a reduction in pH [2]. Due to the rapid fermentation of NSC, volatile fatty acid (VFA) and lactic acid accumulate in the rumen, causing the pH to decrease [4]. If the pH decreases below 5.6, acid-resistant bacteria such as *Lactobacillus* spp. become dominant in the rumen and this environment is disrupted [3]. In this state of lactic acidosis, the host ruminant may experience intake depression, reduced fiber digestion, milk fat depression, diarrhea, ruminitis, lameness, liver abscesses, inflammation, pneumonia, and even death [5]. To prevent or abate the negative effects of increasing the level of concentrates in the diet, a better understanding of the dynamics of the rumen bacterial community is critical.

Numerous studies have reported that the rumen bacterial community is affected by various factors such as species, diet, age, health condition, feed additives, season, and geographical location [2,6]. Several studies have been conducted to characterize the rumen bacterial community in ruminants (e.g., dairy cattle, beef cattle, buffalo, sheep, and goat) fed a HC [7–10]. However, comparative studies on differences among species related to dietary change are scarce, especially for domestic ruminants in Korea. There is one study by Lee et al [11] that compared the rumen microbial communities of three Korean domestic ruminant species, Hanwoo steers (*Bos taurus coreanae*), Holstein-Friesian dairy cattle, and Korean native goats (*Capra hircus coreanae*). Experimental conditions (e.g., diet and management), however, were not controlled in their experiment. In addition, individual variation within each species was not considered.

Therefore, the objective of this study was to investigate the differences in rumen fermentation characteristics and changes in microbial communities associated with the sudden introduction of a HC in three ruminant species. The three ruminants selected for this study were Hanwoo cows, Holstein cows, and Korean native goats. Rumen fermentation characteristics (i.e., pH, NH_3_-N, and VFAs) were determined and two culture-independent techniques, terminal restriction fragment length polymorphism (T-RFLP) and real-time quantitative polymerase chain reaction (PCR), were used to analyze the diversity of rumen microbial communities.

## MATERIALS AND METHODS

### Animal care

This study was conducted at the Center for Animal Science Research, Chungnam National University, Korea. The protocols for this experiment were reviewed and approved by the Chungnam National University Animal Research Ethics Committee (CNU-00455).

### Animals and diets

Four non-pregnant, non-lactating Hanwoo cows (546±33.6 kg), four non-pregnant, non-lactating Holstein cows (516±42.7 kg), and four Korean native goats (19±1.4 kg), individually housed, were used in this 3-week experiment. All animals were fed the same diet composed of timothy hay and a commercial concentrate mix (CM). The chemical compositions of the timothy hay and CM are presented in Table 1. The diet was fed *ad libitum* twice daily at 0800 and 1800. During the first two weeks, a mixed ration with 800 g/kg timothy hay and 200 g/kg CM was offered. On the following week, only the CM was fed to the animals to induce acidosis-like conditions in the rumen. Drinking water was freely accessible by animals throughout the experimental period.

### Collection and preparation of rumen fluid

Rumen fluid was sampled twice (after the 2-week period of high forage feeding on day 14 and after the 1-week period of high concentrate feeding on day 21). Samples were collected using an oral stomach tube 2 h after each morning feeding as described by Duffield et al [12]. Briefly, after initially obtained rumen fluid (approximately 200 mL) was discarded, 500 mL of rumen fluid was collected in a glass flask. The pH of the rumen fluid was analyzed immediately after its collection using a general purpose pH meter (EcoMet P25, Istek, Inc., Seoul, Korea), and then the rumen fluid was transferred to the laboratory. Fifty milliliters of rumen fluid was stored at −20°C until DNA was extracted. Ten milliliters of the rumen fluid was centrifuged at 14,000×*g* for 10 min at 4°C, and two 1-mL aliquots of the supernatants were each transferred to a 2-mL Eppendorf tube and stored at −20°C until VFAs and NH_3_-N concentrations were analyzed.

### Analysis of rumen fermentation characteristics

The VFA concentration was determined as described by Erwin et al [13]. Briefly, 1 mL of rumen fluid supernatant was mixed with 0.2 mL of metaphosphoric acid (250 g/L) and held at 4°C for 30 min. After re-centrifugation of the mixture at 14,000×*g* for 10 min at room temperature, the supernatant was injected into a gas chromatograph (HP 6890, Hewlett-Packard CO., Palo Alto, CA, USA) equipped with a flame ionization detector and capillary column (Nukol fused silica capillary column 30 m× 0.25 mm×0.25 μm, Supelco, Inc., Bellefonte, PA, USA). The temperature of the oven, injector, and detector were 90°C to 180°C, 185°C, and 210°C, respectively. Nitrogen was used as the carrier gas at a flow rate of 40 mL/min. The NH_3_-N concentration was analyzed by the method of Chaney and Marbach [14]. After re-centrifuging the stored rumen fluid at 20,000×*g* for 15 min, 20 μL of the supernatant was mixed with 1 mL of phenol color reagent and 1 mL of alkali–hypochlorite reagent. The mixture was then incubated in a 37°C water bath for 15 min. After the incubation, 8 mL of distilled water was added to the mixture and the NH_3_-N concentration was determined by measuring the absorbance at 630 nm with a spectrophotometer (UV-1800, Shimadzu Inc., Kyoto, Japan).

### DNA extraction

The DNA was extracted from each rumen fluid sample as described by Rius et al [15]. Each 25 mg of homogenized and freeze-dried rumen fluid was added to a bead beating vial with screw cap containing a 0.7-g baked 0.1 mm diameter zirconia/silica bead, 200 μL of 20% sodium dodecyl sulfate solution 282 μL buffer A (NaCl 0.2 M, Tris 0.2 M, ethylenediaminetetraacetic acid 0.02 M; pH 8.0), 268 μL buffer PM (QIAquick 96 PCR Purification Kit, Qiagen Inc., Valencia, CA, USA), 550 μL phenol/chloroform/isoamyl alcohol mixture (25:24:1, vol:vol:vol; pH 8.0) and bead beating for 4 min at full speed using a Mini-Beadbeater-96 (BioSpec Products Inc., Bartlesville, OK, USA). After centrifugation (20,000×*g* for 20 min at 4°C), 500 μL of supernatant was transferred into a 1.5-mL microcentrifuge tube, mixed with 650 μL of buffer PM (Qiagen, USA), and then applied to a column (QIAquick 96 PCR Purification Kit, USA) to bind the extracted DNA. After washing bound DNA with 700 μL of buffer PE (Qiagen, USA), the DNA was eluted into 1.5-mL microcentrifuge tubes using 50 μL of elution buffer (10 mM Tris, pH 8.5 with HCl).

### Terminal restriction fragment length polymorphism analysis

To determine differences in microbial composition among rumen samples, a T-RFLP analysis was performed on the amplified 16S rRNA fragment of the DNA extracted from rumen fluid as described by Fernando et al [7]. Using 100 ng of extracted DNA, the 16S rRNA gene was PCR amplified using Takara Ex Taq (Takara Bio Inc., Shiga, Japan) and a Takara PCR Thermal Cycler Dice Gradient (Takara Bio Inc., Japan). The primers used were a 6-carboxyfluorescein (6-FAM)-labeled forward primer (primer FAMBacT0008F, 5′-AGAGTTT GATCCTGGCTCAG-3′) and an unlabeled reverse primer (primer BacT0805R, 5′-GGACTACCAGGGTATCTAATCCC -3′). The 50-μL PCR mixture contained 2 μL of DNA (100 ng), 0.25 μL of Takara Ex Taq polymerase, 5 μL of 10× Ex Taq buffer, 4 μL of deoxynucleoside triphosphate (dNTP) mixture (2.5 mM), 2 μL each of forward and reverse primers, and 35 μL of sterile distilled water. The cycling conditions were 1 cycle of 1 min at 95°C, 30 s at 52°C, and 1 min at 72°C, followed by 34 cycles of 30 s at 95°C, 30 s at 52°C, and 1 min at 72°C, and a final extension step of 3 min at 72°C. The PCR amplicon was digested by the restriction enzymes HaeIII (Takara Bio Inc., Japan), MspI, and RsaI (Enzynomics, Daejeon, Korea), and then sequenced using a BigDye Terminator v3.1 Cycle Sequencing Kit (Applied Biosystems Inc., Foster City, CA, USA) and ABI 3730XL DNA Analyzer (Applied Biosystems Inc., USA). During the T-RFLP experiment, one of the samples of a Hanwoo cow was damaged; therefore, we were only able to obtain data from three Hanwoo cows.

The T-RFLP data (size, base pair, and peak area for each T-RF) were analyzed as described by Abdo et al [16]. All T-RFLP electropherograms were visually inspected, and the size and peak heights were tabulated using Peak Scanner Software (Applied Biosystems Inc., USA). T-RFs less than 27 bp and greater than 520 bp were discarded. True peaks were identified based on peak heights. Briefly, the standard deviation of the heights of total peaks was calculated assuming that the true mean of the background equaled zero. Peaks that had heights greater than three standard deviations were identified and defined as true peaks. After removing the true peaks from the dataset, the identification process was repeated until no peaks with heights greater than three standard deviations were present in the dataset.

The sizes of the fragments in a sample were determined also by the method proposed by Abdo et al [16]. Data were binned after pooling all fragment lengths of true peaks. After sorting the fragment lengths, duplicate lengths in multiple samples were identified and eliminated. These duplicated fragment lengths were then binned and represented by their average length. Peak heights of binned fragments from the same sample were summed. For each sample, peak heights were standardized by dividing peak heights of fragments in a bin by the sum of peak heights within the sample. The standardized peak heights thus represented the relative abundance of each fragment. The pooled T-RFLP peak heights were used to calculate the Shannon index and the inverse Simpson index as estimates of the diversity of microbial communities in each sample using an R package, Vegan.

### Real-time polymerase chain reaction

Relative quantification of the ruminal bacteria from each rumen fluid sample was performed using quantitative real-time PCR (qPCR) with primers targeting the 16S rRNA gene of different rumen microbial species (Table 2). Real-time PCR amplification was performed on a Bio-Rad CFX 96 Connect Real-Time PCR Detection System (Bio-Rad Laboratories Inc., Hercules, CA, USA), and data were analyzed using Bio-Rad CFX Manager software (version 3.0). Reactions were performed in triplicate in a total volume of 20 μL in 96-well Hard-Shell PCR plates (Bio-Rad, USA) sealed with PCR film (PCR Sealers Microseal ‘B’, Bio-Rad Laboratories Inc., USA). The reaction mixture (per well) contained 10 μL of SYBR Green Real-Time PCR Master Mix (Toyobo Co., Ltd., Osaka, Japan), 1 μL each of forward and reverse primers, 2 μL of DNA template from a rumen fluid sample, and 6 μL of PCR grade water. The PCR cycling conditions were an initial denaturation step at 95°C for 1 min, followed by 40 cycles of denaturation at 95°C for 15 s, annealing at 60°C for 15 s, and extension at 72°C for 45 s. Finally, a melting curve analysis was performed by slowly cooling the reaction mixture from 95°C to 65°C to detect nonspecific amplification products. All reactions were performed in triplicate. The abundance of each target bacteria was calculated relative to the total rumen bacteria population according to the equation 2^−ΔCt^ = 2^−(Ct target−Ct total bacteria)^, as described by Denman and McSweeney [17]. The relative abundance of bacteria was expressed as a percentage of total rumen bacteria (100×2^−ΔCt^).

### Statistical analysis

Data for rumen parameters and the relative quantities of rumen microbial species were analyzed using the MIXED procedure in SAS version 9.4 (SAS Institute Inc., Cary, NC, USA). Animal, nested within species, was included in the model as a random variable. Degrees of freedom were adjusted using KENWARDROGER. A variance component was used in the variance-covariance matrix. The linear model used for the analysis was as follows:

$$y_{ijkl}=\mu +\tau_{i}+b_{j(i)}+\rho_{k}+{\left(\tau \rho \right)}_{ik}+e_{ijkl}$$

Where: *y_ijkl_* is the kth observation in the ith ruminant species and jth diet, μ is the overall mean, *τ_i_* is the fixed effect of the ith species (i = 1 to 3), *b_j(i)_* is the random effect of the jth animal within the ith species, *ρ_k_* is the fixed effect of the kth diet (i = 1 to 2), (*τ_ρ_*)*_ik_* is the interaction between species and diet, and *e_ijkl_* is the unexplained random error.

Differences among means were also compared by Tukey’s test when there was a significant overall effect. Statistical significance was set at p<0.05, and 0.05≤p<0.1 was considered a trend.

A complete-linkage hierarchical cluster analysis (HCA) was performed on the T-RFLP profiles using the hclust function in R version 3.0.2 (http://www.r-project.org/). Euclidean distances were used to generate a distance matrix. A dendrogram of clusters was generated using an R package, dendextend. A principal component analysis (PCA) was also performed without any further normalization to reduce the dimensionality of explanatory variables (i.e., fragment lengths) using the prcomp function in R. PCA results extracted from T-RFLP profiles were plotted with the first principal component on the x-axis and the second principal component on the y-axis.

## RESULTS

### Rumen fermentation characteristics

A rumen acidosis-like condition was achieved in each of the Korean domestic ruminant species by providing only CM (Table 3). The mean ruminal pH decreased, on average, from 6.34 to 5.53 within one week of introducing this diet (p<0.01). The extent of this reduction, however, differed by species (p< 0.01). The decrease in pH units was 1.23 in Holstein cows, while it was only 0.3 in goats. The ruminal NH_3_-N concentration showed a tendency to increase 1.92-fold, on average, after the introduction of the HC (p = 0.096).

A significant interaction between species and diet was apparent in the VFA concentrations (p<0.01). A greater increase in total VFA concentration with the change in diet was observed in Holstein cows (63.15 to 121.62 mM, 1.93-fold) than in the other two species. With the diet change, the proportion of acetate significantly decreased, while that of butyrate and valerate significantly increased in all species (p<0.01). The acetate to propionate (A/P) ratio significantly decreased with the diet change overall (p<0.01), but significant differences between species were also observed (p = 0.049). The A/P ratio remained the same in goats (2.6 vs 2.6), despite the shift to a HC. This was related with no change in the proportion of propionate in goats although it was increased in the other species, which leaded to a tendency toward an interaction between species and diet (p = 0.066). A significant interaction between species and diet was observed in the proportion of branch-chained VFAs (i.e., isobutyrate and isovalerate). The molar proportion of branch-chained VFAs decreased with the diet change in Holstein cows, but it remained the same in Hanwoo cows and even tended to increase in goats (p<0.05).

### Diversity in rumen microbial communities

There were significant differences between species in the diversity of rumen microbial communities (p = 0.022); however, the change to a HC did not affect microbial diversity in the rumen (Table 4). Higher microbial diversity was observed in Korean native goats compared with the two cattle species, based on the Shannon diversity index and Inverse-Simpson index. The diversity indices were significantly lower in Holstein cows than that in goats (p<0.05).

**Figure 1:**
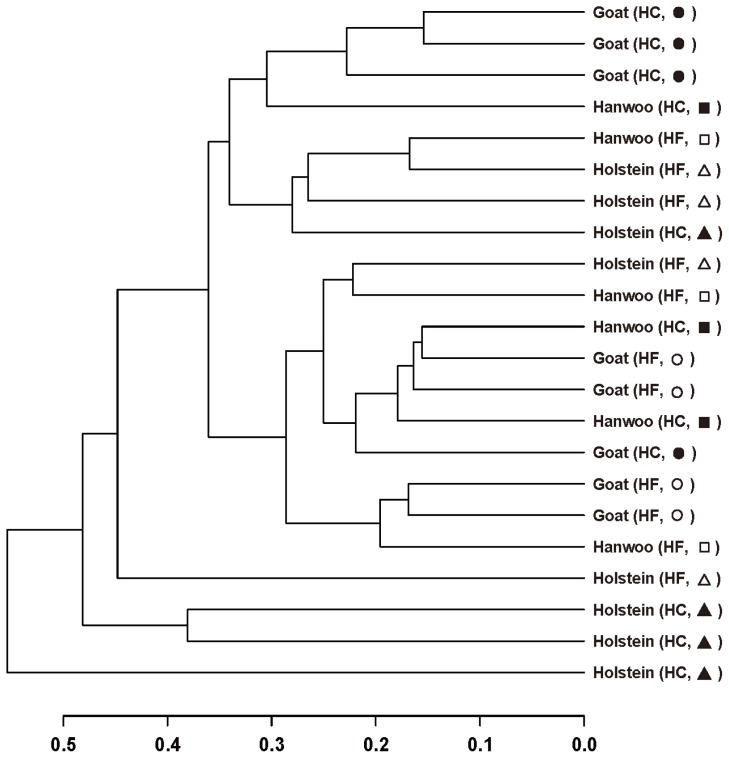
Hierarchical cluster analysis of the similarity of microbial communities in the rumens of Korean domestic ruminants fed a high forage (HF) vs a high concentrate diet (HC) using terminal restriction fragment length polymorphism data. A dendrogram generated by calculating the Euclidean distance between normalized peak heights. Symbols are as follows: Hanwoos fed a high forge (HF, □) or a high concentrate (HC, ■) diet; Holsteins fed a high forage (HF, Δ) or a high concentrate (HC, ▲) diet; goats fed a high forage (HF, ○) or a high concentrate (HC, ●) diet.

**Figure 2:**
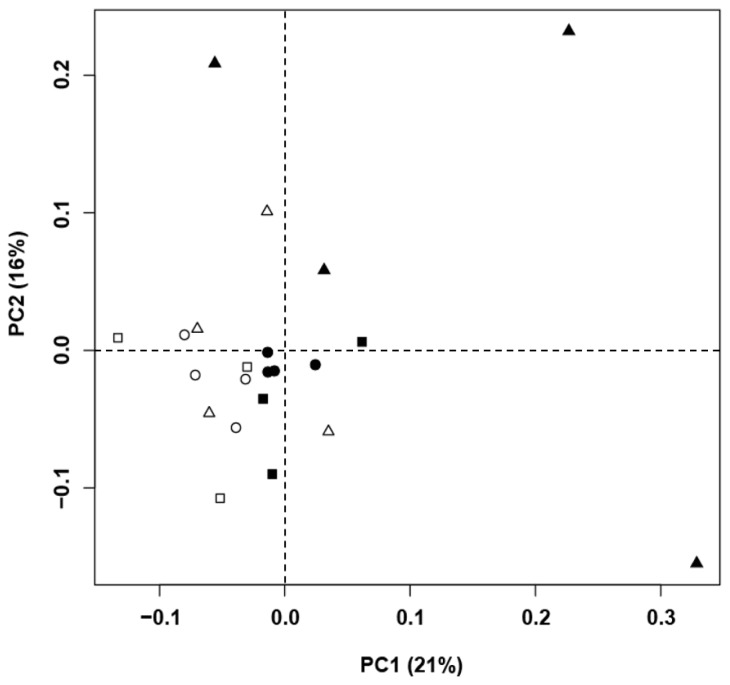
Principal component analysis of the similarity of microbial communities in the rumens of Korean domestic ruminants fed a high forage (HF) vs a high concentrate diet (HC) using terminal restriction fragment length polymorphism data. The first principal component (PC1, x-axis) explained 21% and the second principal component (PC2, y-axis) explained 16% of the total variance. Symbols are as follows: Hanwoos fed a high forge (HF, □) or a high concentrate (HC, ■) diet; Holsteins fed a high forage (HF, Δ) or a high concentrate (HC, ▲) diet; goats fed a high forage (HF, ○) or a high concentrate (HC, ●) diet.

An HCA of the T-RFLP data was performed to assess a relative homogeneity in rumen bacterial community structures among the different animal species and diets (Figure 1). Although no systematic patterns were observed in the clusters, there was a tendency toward more similar microbial communities among goats and among Hanwoo cows when they were fed a HC. PCA indicated that diet was more important in determining the bacterial community structure than species (Figure 2). The first principal component (PC1) explained 21% of the data variance, and the second principal component (PC2) explained 16% of the data variance.

### Relative population of selected bacterial species

Relative populations of selected bacterial species in the rumen fluid of Korean domestic ruminants were also analyzed using real-time quantitative PCR (Table 5). The relative abundance of *Prevotella* spp. was 42%, on average, and it did not differ by either diet or species.

Among the cellulolytic bacterial species, the relative abundance of *F. succinogenes*, but not *Ruminococcus flavefaciens* (*R. flavefaciens*), significantly decreased with high concentrate feeding (p<0.01). A greater reduction in the proportion of *F. succinogenes* was observed in Korean native goats than in the other animal species (13-fold; p<0.01).

Among major species of lactate producers in the rumen, *Lactobacillus* spp. significantly increased (39-fold; p<0.05), whereas *S. bovis* did not change significantly with the switch to a HC (p>0.1). *S. ruminantium*, known as both a lactic acid producer and utilizer, did not show a significant change with the diet transition (p>0.1). A great increase (approximately 1,700-fold) in the relative proportion of *M. elsdenii*, a major lactate utilizer, was observed (p<0.01) in all experimental animal species fed a HC.

## DISCUSSION

All ruminants interact closely with rumen microorganisms. The rumen microbes supply VFAs, proteins, and vitamins to the host ruminant by degrading and fermenting feed materials [1]. The ruminal bacterial community composition can change depending on the type of diet ingested by the ruminant animal [1]. Studies on microbial communities and ruminal characteristics are important for understanding and manipulating the health and performance of ruminant animals. Previous studies have shown that changes in rumen microbial communities are affected by factors such as ruminant species, diet, age, health, feed additives, season, and geographical location [2,6,18,19]. In particular, a diet switch from high forage to high concentrate can cause a large change in the rumen bacterial community, which can affect productivity and the potential for metabolic disease in ruminants [20]. In general, a HC increases the number of both lactic acid producers and utilizers and, while the number of fiber-degrading bacteria drastically decrease because of the low fiber content and reduction in pH resulting from the accumulation of lactic acid in the rumen [10]. Because of changes in the bacterial community, total VFA and lactic acid concentrations in the rumen increase [4]. When the pH decreases below 5.6, *Lactobacillus* spp., more acid-resistant than other microbes, become dominant in the rumen, which can cause metabolic disorders [4]. Despite the fact that it is important to understand the changes in the rumen microbial communities resulting from supplying a HC, little research has been done on the microbial communities of ruminant species in Korea. In the present study, changes in the rumen condition and microbial communities by supplying a HC were investigated in three ruminant species (Hanwoo, a major beef breed; Holstein, a major dairy breed; Korean native goat) commonly raised in the intensive farming system in Korea. They were housed in similar conditions and fed the same diet.

Based on the proposed pH thresholds to define the ruminal acidosis when rumen fluid samples are collected by using an oral stomach tube [12,21], it seemed that Hanwoo cows were experienced a subacute ruminal acidosis. Also, Holstein cows seem to had an acute ruminal acidosis-like condition because the pH of rumen fluid below 5.2 unit is considered to be the development of acute ruminal acidosis [22]. According to the those threshold, Korean native goats also showed subacute ruminal acidosis condition, however, the pH change of Korean native goats were no significant after introduction of HC compared to the cattle species. It seemed that Holstein cows were more susceptible while Korean native goats more resistant to ruminal acidosis. After providing only the CM, there were a dramatic increase in total VFA concentration and a significant reduction in pH in Holstein cows. In the goats, however, no such changes were observed. A reduction in ruminal pH is closely related with the concentration of total VFAs. Previous studies showed an increase in the concentration of VFA rather than lactate primarily affected the ruminal pH in response to high concentrate feeding in cattle [23]. In our study, an increase in total VFAs was observed in both cattle species. Changes in relative proportions of individual VFAs followed a typical response to an acidosis-like condition in the rumen [4,24]. However, total VFA concentration of Korean native goats did not increase with the feed change and thus the ruminal pH was little reduced. A week of feeding a HC may not be sufficient to induce ruminal acidosis in Korean native goats. Previous studies have indicated Korean native goats may be resistant to reduction in ruminal pH by dietary changes. Even feeding a HC (800 g/kg CM and 200 g/kg forage) for 21 days was not able to reduce the ruminal pH of Korean native goats less than 6.0 [25]. Although Lee et al [11] could induce acidosis like condition (pH<5.11) in Korean native goats by feeding a diet composed of 900 g/kg CM and 100 g/kg forage, they did not report the duration of feeding the diet.

Early studies on the rumen ecosystem used culture-based methods to identify microorganisms [1,26]. However, due to the fastidious growth requirements of many rumen microbes, culture-based methods have important limitations for the assessment of microbial changes, namely, they substantially underestimate microbial diversity in the rumen and fail to detect major microbes. Therefore, studies on changes in microbial diversity in the rumen now use molecular techniques. Whitford et al [27] was the first to analyze the phylogenetic diversity of rumen microbial communities based on 16s rRNA sequences, and to demonstrate that many microbes present in the rumen were difficult to culture. Tajima et al [20] reported a rumen bacterial transition with a change from roughage to a high-grain diet in Holstein cows, based on a 16S rRNA sequence analysis. Since then, a variety of molecular analytical methods have been developed and used to evaluate this microbial transition. T-RFLP analysis, denaturing gradient gel electrophoresis (PCR-DGGE), real-time PCR, and pyrosequencing methods have been widely used in recent studies on dairy cattle, beef cattle, sheep, and goats [7–10].

In our study, we determined changes in the structure and diversity of bacterial communities in the rumen using T-RFLP analysis, as well as changes in specific bacterial species using real-time PCR analysis. The Shannon index and Inverse-Simpson Index calculated based on the results from a T-RFLP analysis indicated diversity of bacterial community in the rumen were more diverse in goats compared with Holstein cows. This is the first study that directly compared the diversity of bacterial community between Korean native goats and Holstein cows, and further study is warranted to decipher the differences in the microbial communities between two species. In addition, the diversity of bacterial community was not altered by a HC, which was consistent with previous studies [8,28,29]. It may indicate that feeding a HC for a week did not change the diversity but the structure of bacterial community in the rumen.

The changes in the structure of bacterial community in the rumen by feeding a HC were supported by the results from HCA and PCA analyses. HCA and PCA showed a tendency of clustering of ruminal bacterial communities more by diet than species, and suggest that rumen bacterial community structure was influenced by the HC. Comparative analysis using a real-time PCR clearly showed changes in the structure of bacterial community in the rumen by feeding a HC. Real-time PCR analysis is a powerful method that can be used to quantify specific target DNA sequences [30]. We confirmed significant changes in the relative populations of *F. succinogenes*, *Lactobacillus* spp., and *M. elsdenii* among the seven major bacterial species.

A 12-fold decrease, on average, in the proportion of *F. succinogenes* in all animals was observed in this study. *F. succinogenes* is one of the dominant cellulolytic bacteria species in rumen [31]. A reduction of *F. succinogenes* following the switch from a high forage diet to HC was also noted in some previous reports. Tajima et al [10] showed that the proportion of *F. succinogenes* decreased 20-fold on day 3 and decreased 57-fold on day 28 in animals after a change to a HC. A 40-fold reduction in this population with the introduction of a HC was also reported by Fernando et al [7]. On the other hand, there was no significant change in the *R. flavefaciens* population in the present study, which was consistent with a previous study [32]. The reason for this is unclear, but *R. flavefaciens* showed high resistance to low rumen pH than *F. succinogenes*.

As agreed with previous studies, the proportion of *Lactobacillus* spp. was drastically increased after feeding a HC. On the contrary, there was a reduction in the population of *S. bovis* with persistent acidity in the rumen, which was consistent with other studies [7,10,33]. *S. bovis* and *Lactobacillus* spp. are known as typical lactic acid-producing bacteria, and they are primarily responsible for reducing the pH in the rumen [34]. Generally, the ruminal population of *S. bovis* is increased when animals were fed a diet composed of a highly fermentable carbohydrate source such as glucose or starch. However, as the ruminal pH becomes more acidic (<6.0), its growth diminished, and *Lactobacillus* spp., more tolerant to low pH than *S. bovis*, becomes dominant in the acidic rumen when pH is less than 5.6 [35].

With the accumulation of lactic acid in the rumen resulting from a HC, the numbers of bacteria that utilize lactic acid increase [36]. *M. elsdenii* and *S. ruminantium* are the predominant lactic acid utilizers in the rumens of animals fed a HC [36,37]. *M. elsdenii* uses 60% to 80% lactic acid in the rumen; therefore, *M. elsdenii* helps prevent accumulation of lactic acid in the rumen [38]. In this study, the *M. elsdenii* proportion drastically increased relative to other major bacteria with the change to a HC. Fernando et al [7] also reported an increase in the *M. elsdenii* proportion, based on a real-time PCR analysis. While the proportion of *S. ruminantium* did not change significantly in this study, some studies have reported otherwise. Tajima et al [10] reported an approximately 9-fold increase in this species in animals fed a HC. Similarly, Fernando et al [7] reported a 30-fold increase the *S. ruminantium* proportion. However, some recent studies showed similar results to our current study which were report that there was no significant change in population of *S. ruminantium* when ruminal acidosis was induced [39,40]. There have been reports that *S. ruminantium* grows slowly in acidic rumen [41] and is less able to use lactic acid than *M. elsdenii*, because its lactic dehydrogenase is repressed by glucose and maltose [42]. These results may support findings in the current study.

In summary, based on the results from the analyses of rumen fermentation characteristics and rumen bacterial community structure, differences between the three animal species fed a HC were apparent. Based on rumen fermentation characteristics, both cattle species showed signs of acidosis. In particular, Holstein cows showed greater sensitivity to the concentrate diet than the other two native Korean species, exhibiting signs of severe acidic (acute acidosis-like) conditions. Further, we were able to confirm that there is a change in the rumen bacterial community structure, and that the most significant change occurred in the Holstein cow. Results of the real-time PCR analysis of the major bacteria associated with acidosis showed a reduction in cellulolytic bacteria and an increase in lactic acid producers and utilizers. Unlike the structure of bacterial community, the bacterial diversity was not affected by feeding a HC for a week. The diversity of bacterial community, however, significantly differed by ruminant species. To determine why acidosis does not progress in the rumens of native Korean goats and to precisely determine the degree of rumen acidosis within each ruminant species, continuous monitoring of pH and lactate concentrations in rumen beginning with the introduction of a HC will be needed. Because of the considerable variability between animals within the same species, the number of experimental animals in each species should be increased. Despite the differences in rumen fermentation characteristics and rumen bacterial community structure observed between animal species, the real-time PCR analysis result could not refine these differences. Therefore, to better identify the changes in bacterial populations in an acidified rumen, the classification level of target bacteria should be expanded from species to genus and family.

## Figures and Tables

**Table 1:** Chemical composition (g/kg DM or as stated) of the ruminant diet

Item	Timothy hay	Concentrate mix1)
DM (g/kg as fed)	887	864
OM	924	938
CP	75	149
SOLP	25	48
NDICP	13	22
ADICP	12	15
aNDF	657	245
ADF	450	124
ADL	68	33
Ether extract	14	40
Ash	76	62
TDN	535	766
NEm (MJ/kg DM)	5.0	7.6
NEg (MJ/kg DM)	2.6	5.0
Total carbohydrates	835	750
NFC	191	526
Carbohydrate fractions (g/kg carbohydrate)		
CA	117	115
CB1	9	536
CB2	103	51
CB3	574	194
CC	196	104

DM, dry matter; OM, organic matter; CP, crude protein; SOLP, soluble CP; NDICP, neutral detergent insoluble CP; ADICP, acid detergent insoluble CP; aNDF, neutral detergent fiber analyzed using a heat stable amylase and expressed inclusive of residual ash; ADF, acid detergent fiber; ADL, acid detergent lignin; TDN, total digestible nutrients; NEm, net energy for maintenance; NEg, net energy for growth; NFC, non-fiber carbohydrate; CA, carbohydrate A fraction, ethanol soluble carbohydrates; CB1, carbohydrate B1 fraction of starch; CB2, carbohydrate B2 fraction of soluble fiber; CB3, carbohydrate B3 fraction of available insoluble fiber; CC, carbohydrate C fraction of unavailable carbohydrate.

1) Concentrate mix (g/kg DM): 249 corn flakes, 186 ground corn, 96 ground wheat, 35 rice bran, 69 gluten feed, 43 wheat flour, 18 dried distillers grains with solubles, 84 palm kernel meal, 60 copra meal, 49 cottonseed hulls, 45 molasses, 11 palm olein oil, 24 limestone, 6 salt, 3 magnesium oxide (50%), 1 sodium bicarbonate, 5 ammonium chloride, 13 CMS/MSG, 2 rapeseed meal, 1 vitamin and mineral mix.

**Table 2:** Primer sets used for relative quantification of bacterial species in the rumen using real-time polymerase chain reaction

Bacterial species	Primer sequence (5′-3′)	Product size (bp)	Reference
Total bacteria	F: CGGCAACGAGCGCAACCC	130	Denman and McSweeney [17]
	R: CCATTGTAGCACGTGTGTAGCC		
*Prevotella* spp.	F: GGTTCTGAGAGGAAGGTCCCC	121	Stevenson and Weimer [43]
	R: TCCTGCACGCTACTTGGCTG		
*Fibrobacter succinogenes*	F: GTTCGGAATTACTGGGCGTAAA	121	Denman and McSweeney [17]
	R: CGCCTGCCCCTGAACTATC		
*Ruminococcus flavefaciens*	F: CGAACGGAGATAATTTGAGTTTACTTAGG	132	Denman and McSweeney [17]
	R: CGGTCTCTGTATGTTATGAGGTATTACC		
*Streptococcus bovis*	F: TTCCTAGAGATAGGAAGTTTCTTCGG	82	Stevenson and Weimer [43]
	R: ATGATGGCAACTAACAATAGGGGT		
*Lactobacillus* spp.	F: AGCAGTAGGGAATCTTCCA	345	Lan et al [44]
	R: ATTCCACCGCTACACATG		
*Selenomonas ruminantium*	F: CAATAAGCATTCCGCCTGGG	71	Stevenson and Weimer [43]
	R: TTCACTCAATGTCAAGCCCTGG		
*Megasphaera elsdenii*	F: GACCGAAACTGCGATGCTAGA	129	Ouwerkerk et al [45]
	R: CGCCTCAGCGTCAGTTGTC		

**Table 3:** Rumen fermentation characteristics in Korean domestic ruminants fed a high forage (HF) vs a high concentrate diet (HC)

Variables	Hanwoo		Holstein		Goat		SEM	p-value		
			
	HF1)	HC2)	HF	HC	HF	HC		Species	Diet	Species×diet
pH	6.39^a^	5.56^bc^	6.37^a^	5.14^c^	6.25^a^	5.90^ab^	0.121	0.119	<0.01	<0.01
NH_3_-N, (mg/dL)	1.27	4.97	2.56	4.84	5.19	7.51	1.890	0.264	0.096	0.908
Total VFA (mM)	74.3^b^	81.5^b^	63.1^b^	121.6^a^	59.4^b^	53.8^b^	6.81	<0.01	<0.01	<0.01
VFA (mmol/mol)										
Acetate	590^a^	470^b^	585^a^	464^b^	562^a^	478^b^	13.2	0.779	<0.01	0.310
Propionate	175	272	170	210	218	198	23.4	0.383	0.054	0.066
Butyrate	123^b^	130^ab^	118^b^	215^a^	88^b^	164^ab^	18.1	0.093	<0.01	0.071
Isobutyrate	59^ab^	55^ab^	68^ab^	38^b^	71^a^	82^a^	6.3	0.011	0.145	0.026
Valerate	28^c^	46^ab^	31^bc^	55^a^	34^bc^	44abc	4.2	0.480	<0.01	0.199
Isovalerate	25^ab^	28^ab^	29^ab^	18^ab^	27^ab^	35^a^	3.3	0.105	0.954	0.024
A:P ratio	3.4^a^	1.8^b^	3.5^a^	2.5^ab^	2.6^ab^	2.5^ab^	0.29	0.316	<0.01	0.049

SEM, standard error of the mean; VFA, volatile fatty acid.

1) HF, high forage diet; TMR with 800 g/kg timothy hay and 200 g/kg concentrate mix.

2) HC, high concentrate diet; only the concentrate mix.

a–c Means that do not have common superscripts significantly differ within the species (p<0.05).

**Table 4:** Diversity of the microbial communities in rumens of Korean domestic ruminants fed a high forage (HF) vs a high concentrate diet (HC) using terminal restriction fragment length polymorphism data

Index	Species1)					Diet2)				Species×diet
		
	Hanwoo	Holstein	Goat	SEM	p-value	HF	HC	SEM	p-value	p-value
Shannon index	2.73^ab^	2.56^b^	2.92^a^	0.100	0.022	2.72	2.76	0.075	0.691	0.407
Inverse-simpson index	11.78^ab^	9.74^b^	14.67^a^	1.292	0.022	11.44	12.69	0.963	0.373	0.632

SEM, standard error of the mean.

1) Hanwoo, Korean native cow; Holstein, Holstein cow; Goat, Korean native goat.

2) HF, high forage diet, TMR with 800 g/kg timothy hay and 200 g/kg concentrate mix; HC, high concentrate diet, only the concentrate mix.

a,b Means that do not have common superscripts significantly differ within the species (p<0.05).

**Table 5:** Relative abundance (% of total bacteria) of selected bacterial species in the rumens of Korean domestic ruminants fed a high forage (HF) vs a high concentrate (HC) diet

Bacterial species	Hanwoo		Holstein		Goat		SEM	p-value		
			
	HF1)	HC2)	HF	HC	HF	HC		Species	Diet	Species×diet
*Prevotella* spp.	37.921	40.747	41.885	37.905	39.599	51.827	3.7177	0.193	0.240	0.120
*F. succinogenes*	0.774^ab^	0.039^b^	0.819^ab^	0.099^b^	2.187^a^	0.171^b^	0.3642	0.087	<0.01	0.153
*R. flavefaciens*	0.010	0.025	0.016	0.001	0.008	0.003	0.0081	0.296	0.801	0.186
*S. bovis*	2.715	0.020	0.782	0.011	0.158	0.015	0.9344	0.380	0.132	0.383
*Lactobacillus* spp.	0.016	0.380	0.040	1.041	0.002	0.862	0.3862	0.680	0.041	0.691
*S. ruminantium*	3.475	3.037	3.632	3.169	5.812	3.902	1.4698	0.497	0.445	0.850
*M. elsdenii*	0.001^b^	1.450^ab^	0.002^b^	3.402^a^	0.001^b^	1.692^ab^	0.7372	0.373	<0.01	0.374

SEM, standard error of the mean.

1) HF, high forage diet, TMR with 800 g/kg timothy hay and 200 g/kg concentrate mix.

2) HC, high concentrate diet, only the concentrate mix.

a,b Means that do not have common superscripts significantly differ within the species (p<0.05).
